# Iodine Status in the Colombian Population and the Impact of Universal Salt Iodization: A Double-Edged Sword?

**DOI:** 10.1155/2019/6239243

**Published:** 2019-04-01

**Authors:** Hernando Vargas-Uricoechea, María Virginia Pinzón-Fernández, Beatriz Eugenia Bastidas-Sánchez, Elisa Jojoa-Tobar, Luis Eduardo Ramírez-Bejarano, Julián Murillo-Palacios

**Affiliations:** ^1^Director of the Metabolic Diseases Study Group, Associate Professor of the Department of Internal Medicine, Universidad del Cauca, Popayán, Cauca, Colombia; ^2^Full Professor, Department of Internal Medicine, Universidad del Cauca, Popayán, Cauca, Colombia; ^3^Specialist in Family Health and Epidemiology, Full Professor, Department of Social Medicine and Family Health, Universidad del Cauca, Popayán, Cauca, Colombia; ^4^Full Professor, Department of Nursing, Universidad del Cauca, Popayán, Cauca, Colombia; ^5^Metabolic Diseases Study Group, Universidad del Cauca, Popayán, Cauca, Colombia

## Abstract

Iodine deficiency and iodine excess have severe consequences on human health and have been associated with the presence of goiter, hypothyroidism, hyperthyroidism, thyroid cancer, thyroid nodules and thyroid autoimmunity, poor mental health, and impaired intellectual development. Universal salt iodization programs have been considered one of the most cost-effective interventions for the prevention of iodine deficiency-associated disorders, as evidenced over time since the implementation of such programs. However, these efforts have also led to an excessive consumption of iodine in certain geographical regions, due to salt overuse. Consequently, the amount of iodine derived from salt intake exceeds the established limits required for achieving the right balance between salt consumption and health benefits and leads to undesirable health effects. In Colombia, the recommendations and standards for the production and commercialization of iodized salt are fully complied with. Nevertheless, there is a remarkable rate of iodine excess among the country's population, which, at least hypothetically, represents a higher risk for developing functional and structural disorders of the thyroid gland. This review analyzes universal salt iodization programs worldwide, particularly their impact on the thyroid gland and the results of the studies conducted in Colombia following the implementation of such strategy.

## 1. Introduction

Iodine intake-associated health disorders continue to represent major public health issues around the world. Historically, iodine deficiency disorders (IDDs) have been extensively studied. Considered to be of major relevance for their long-recognized pathologies, the IDDs have received considerable attention from government regulators as well [1, 2]. Iodine deficiency is a constant natural phenomenon, affecting populations worldwide. Populations living in iodine-deficient areas are subject to associated pathologies (increased perinatal mortality, mental retardation, hypothyroidism, and endemic goiter) and poor socioeconomic development.

International preventive initiatives have resulted in a fundamental change in the prevalence of IDDs; however, there are still around two billion people at risk of experiencing such disorders. In iodine-deficient areas, universal salt iodization (USI) is the most cost-effective strategy to control these disorders [3, 4]. Salt, as an ingredient used globally and at relatively constant rates throughout the year, is the preferred vehicle to supply iodine to a target population. Adding small amounts of iodine to the nationwide salt supply allows for convenient provision of the level necessary to meet the average daily requirements to avoid IDDs. Furthermore, the iodization technology is simple and low cost; consequently, most countries have adopted the USI process as mandatory for human consumption. Nevertheless, the indicators used to monitor the IDDs (process, impact, and sustainability indicators) are not always systematically implemented. This is why the world distribution of these disorders has changed with a significant rise in the geographical areas with a “more than adequate” or “excessive” intake of iodine [5, 6]. Moreover, disorders associated with excessive iodine intake have been linked to thyroid autoimmunity, hypothyroidism, hyperthyroidism, goiter, and thyroid nodular disease, inter alia (Table 1). In 2017, 11 countries were identified with “excessive iodine intake”, and this change in areas that were previously deficient (but now behave as areas with excessive iodine levels) is a predictor for changes in the frequency of other thyroid pathologies [7, 8]. Notwithstanding this fact, it is believed that the health benefits of USI exceed the iodine excess-associated thyroid dysfunction risk resulting from the intervention. The criteria for evaluating iodine nutrition in the population are shown in Table 2.

## 2. Iodine Intake and Its Thyroid Impact

The exact mechanism through which excess iodine results in thyroid dysfunction is unknown. There are several hypotheses, including that excess iodine induces the production of cytokines and chemokines, leading to the recruitment of immunocompetent cells into the thyroid, which, together with the processing of excessive intrathyroid iodine, could induce oxidative stress, thereby increasing lipid oxidation and tissue damage. The inclusion of iodine in thyroglobulin (Tg) has been shown to induce greater Tg antigenicity, thus putting the individual at higher risk of thyroid autoimmunity [9, 10].

The thyroid gland has an intrinsic mechanism of adaptation to excessive iodine. As such, the so-called “acute Wolff–Chaikoff effect” may be explained by the generation of inhibitor substances (namely, iodolactones, iodine aldehydes, and iodine lipids) that impact the thyroid peroxidase (TPO) activity; the subsequent decrease in intrathyroid deiodinases leads to a reduction in the synthesis of thyroid hormones [11, 12]. In most individuals who experience rapid onset of excessive iodine, this effect is transient and recovers upon the occurrence of another phenomenon called “escape” from (or “adaptation” to) the acute Wolff–Chaikoff effect, which is associated with a reduced expression of the sodium-iodide symporter (NIS), a mediator of the active transport system by which iodine is shuttled from the circulation into the thyroid cell.

The decline in the NIS expression presents approximately 24 hours after acute iodine excess; the subsequent reduction in intrathyroid iodine concentration and in the levels of “iodinated” substances inhibits the synthesis of thyroid hormones and—under normal conditions—reinitiates the production of such hormones [13, 14]. In vulnerable individuals, such as those with thyroid antibodies present, individuals with a history of thyroiditis, radioactive iodine users, or individuals on medications such as amiodarone, interferon-*α*, or lithium, inter alia, the Wolff–Chaikoff escape phenomenon may fail, leading to permanent hypothyroidism. Likewise, in susceptible patients, including those with nontoxic nodular goiter, latent Graves' disease (also known as Basedow's), or those residing in areas with extended and severe iodine deficiencies, excess iodine may lead to hyperthyroidism; this condition and its underlying pathology is known as the “Jod-Basedow phenomenon.”

This metabolic disorder is relatively frequent in areas where iodine intake is very high. In fact, in areas where intake is marginal (but iodine deficiency is nonexistent), moderate increases in iodine intake may result in hyperthyroidism in individuals with autonomous nodular thyroid disease [15, 16]. Moreover, an increase in the frequency of well-differentiated thyroid cancer (WDTC) has been reported in some areas where USI programs have been implemented. However, notwithstanding the association identified in some countries between WDTC and the intake of iodized salt, it is also true that there has been an increase in the frequency of this type of cancer in other countries where the use of this type of salt is low. Generally speaking, iodine deficiency is associated with an increased risk of WDTC (particularly the follicular variety) and probably of anaplastic cancer. In contrast, a high iodine intake (particularly in iodine-deficient areas) has been associated with a nonsignificant reduction in the risk of thyroid cancer. In animal models, for example, both excess and deficit of iodine increase the proliferation of thyroid cells and the formation of thyroid adenomas, but do not affect the risk of carcinogenesis. However, upon exposure to radiation, these animals (both those with deficient and excessive iodine status) show increased risk of thyroid cancer, leading to the hypothesis that both excess and deficiency may be considered “promoters” of thyroid cancer rather than “inducers” [17, 18]. Furthermore, iodine deficiency promotes the generation of oxygen reactive species that cause direct DNA damage and apoptosis and increased risk of mutations [19, 20].

Whilst it is not possible to establish a definite “cause-effect” association between the intake of iodine and thyroid cancer, the growing use of diagnostic methods must also be taken into account (ultrasound, CT, and MRI) as well as the indiscriminate use of fine needle aspiration, which have contributed to increased detection of thyroid cancer. For example, in the United States of America, between the years 1975 and 2009, the incidence of this type of cancer rose from 4.9 to 14.3 per 100,000 inhabitants, while the rate of mortality remained constant (from 0.56 to 0.52 per 100,000, respectively) [21, 22]. Such disease-based screening has contributed to “overdiagnosis” and potential “overtreatment,” accounting for the increase in the frequency of thyroid cancer over the last few decades (rather than to the implementation of USI programs).

## 3. Historical and Conceptual Framework of Iodine Status in the Colombian Population

In was not until 1948 that the prevalence of endemic goiter was identified in Colombia, reporting a 53% rate (based on a national sample of 183,243 school children); however, the number was even higher in some states, such as Caldas and Cauca (>80%). Salt iodization for human use was introduced in 1947 at the national level. A salt iodization project was formally introduced in 1950, with a large production directed to the municipalities presenting the highest rates of endemic goiter at that time [23, 24]. The National Institute for Nutrition (INN) evaluated the intervention in 1952, through a study of 6,511 school children, identifying a prevalence of goiter of 33%. Then, in 1955, the content of iodine in salt was regulated between 50 and 100 parts per million (ppm).

In 1960, the INN together with the Interdepartmental Nutrition Committee for National Defense of the United States evaluated 4,818 adults and 1,263 children in Colombia, identifying a prevalence of endemic goiter of 39.5% in males and of 43.7% in females. In 1965, the INN administered a survey to 12,266 school children living in eight municipalities of the Caldas state (where the prevalence had been >80% in 1948), documenting a prevalence of goiter of <2%. The efficacy of the USI intervention on the population was shown through the nationwide health study (1977–1980), resulting in a <2% prevalence of goiter. Unfortunately, in the early 1980s, a salt for human use that failed to meet the standards established by the Ministry of Health was commercialized, leading to an increase in the prevalence of endemic goiter in some regions, such as Chámeza and Yopal (Casanare State) of 52% and 13.5%, respectively.

In August 1992, the Ministry of Health established a committee for the surveillance of IDDs, and a work group involving various academic agencies was appointed, with the objective of designing a research protocol to assess the prevalence of IDDs; the protocol was formally approved in 1993. As a consequence of the open economy model, salt became a free trade good, and henceforth both iodized and noniodized salt (or the mixture of the two) became available in the market. As a result, the prevalence of goiter rose to 15% in 1993 in certain regions of the country (Table 3). In 1994, the National Institute for the Surveillance of Medicines and Food became responsible for developing the national quality control program for salt. And, 2 years later, in 1996, the regulatory procedures for issuing a sanitary registry and controlling the sanitary conditions for production, packaging, and marketing of salt were introduced [25–28]. Furthermore, salt was classified as food, and the requirement of having a proportion of 50–100 ppm iodine (in the form of iodide) was ratified.

In that same year, the 1996–2005 national food and nutrition plan was implemented; the plan defined the strategies for the prevention and control of micronutrient deficiencies, with the intent of improving iodized salt surveillance and control programs. The success of this plan was recognized on April 29, 1998, when several international health agencies (including UNICEF, WHO/PAHO, and the International Council to Control IDDs (ICCIDD)) declared Colombia as an “IDDs-free country.”

## 4. Studies That Assessed IDDs in Colombia following the Declaration as an IDDs-Free Country

In accordance with the research protocol for the evaluation of IDDs approved in 1993, the study on “The Prevalence of IDDs and Average Salt Intake, Colombia 1994–1998” assessed the prevalence of goiter, urinary iodine, and salt iodine content in a national sample of 19,530 children. The prevalence of grade I goiter was found to be 6.5%, with the highest prevalence levels recorded in the States of Santander and Norte de Santander (20.6%), Tolima, Huila, and Caquetá (16.7%), Bogotá (11.2%), Quindío, Risaralda, and Caldas (8.1%), and the Pacific Coast—Quibdó, Buenaventura, and Tumaco (6.2%). 93.6% of the urinary iodine concentrations were >100 *µ*g/L; concentrations of >200 *µ*g/L were specifically discriminated; only 6.4% of urinary iodine concentrations measured <100 *µ*g/L. The average salt intake per person, per day, was 11.42 g, with 69.3% of the national population exhibiting this level of salt intake [29].

Then, in 1998, the sentinel trial reported a mean level of salt iodine of 60 ppm and a mean urinary iodine clearance of >200 *µ*g/L. Finally, sentinel trials were conducted in 1999 and from 2001 to 2002. In 1999, the mean urinary iodine was 380 *μ*g/L, with medians in the rural and the urban areas of 148 and 705 *μ*g/L, respectively; 78.9% of the population exhibited excess iodine intake, with higher levels recorded in urban areas. The 2001–2002 sentinel trial found a median urinary iodine of 415 *μ*g/L, with 430 *μ*g/L in the urban areas and 380 *μ*g/L in the rural areas; at this time, nearly all (95.7%) of the samples tested had a urinary iodine concentration of ≥100 *μ*g/L. 67.5% of the urinary iodine levels were >300 *μ*g/L (both in the rural and in the urban areas), indicating that 85% of the population had an excessive iodine intake [30–32].

A cross-sectional study of school children, aged 8–18 years, in the Quindío State was published in 2009. The urinary iodine levels were evaluated in accordance with the socioeconomic status and the geographical area (urban or rural). The median urinary iodine for the whole population was 272.4 *μ*g/L, with 88.1% of the school children having abnormal urinary iodine levels. These abnormal concentrations represented both iodine deficit and risk of hyperthyroidism due to excess iodine intake; specifically, 28.8% of the school children had urinary iodine levels <100 *μ*g/L, 11.9% had normal urinary iodine levels (100–199 *μ*g/L), and 59.3% had a urinary iodine ranging from 200 to >300 *μ*g/L. Among the iodine-deficient school children, 11.5% were in the severe deficit range (<20 *μ*g/L), 12.6% were in the moderate deficit range (20 to 49 *μ*g/L), and 4.7% were in the mild deficit range (50–99 *μ*g/L). The prevalence of “more than adequate” iodine intake was 16.2%, and the frequency of “excessive intake” of iodine was 43%. The rural municipalities exhibited 100% iodine deficit, while the urban areas exhibited excessive iodine intake [33].

A subsequent cross-sectional study, published in 2010, assessed the frequency of goiter, anti-TPO antibody titers, urinary iodine, and thyroid-stimulating hormone (TSH) in 128 school children (aged 5–15 years) in Bogotá and Chía (Cundinamarca). Among the children, 16% had elevated TSH levels and 4% showed positive anti-TPO titer; the mean urinary iodine was 401.2 *μ*g/L (4.6% of the patients showed levels between 50 and 99 *μ*g/L (mild deficiency) and 72.65% had levels of ≥300 *μ*g/L). Additionally, 88% of the children were documented to exhibit some degree of goiter [34].

Another cross-sectional study published in 2011 (adults >35 years old, in Armenia city) determined the frequency of hypothyroidism and its relationship with anti-TPO antibodies and elevated urinary iodine among 437 individuals (mostly females). 18.5% of the subjects had elevated TSH levels, and 2.1% exhibited low levels; 28.9% had positive anti-TPO. The prevalence of positive anti-TPO among individuals with TSH >10 mIU/L was 44% and among those with TSH between 5.1 and 10 mIU/L was 19.6%. The mean urinary iodine was 424.2 *μ*g/L, and 80% of the individuals showed levels of >300 *μ*g/L. No relationship was found between the levels of urinary iodine and anti-TPO titers [35].

A later cross-sectional study, conducted between 2013 and 2014, showed the nutritional iodine status and thyroid function in 392 healthy pregnant women (14–45 years old) in an urban area in Bogotá. The prevalence of goiter, TSH levels, urinary iodine, and anti-TPO among other parameters were evaluated. The results showed that 25.5% of the pregnant women had some level of goiter, and the prevalence of overt hypothyroidism and of subclinical hypothyroidism was 1.27% and 14%, respectively (using a TSH cutoff of >4.2 mIU/L as the upper limit of normal and of >10 mIU/L for diagnosing overt hypothyroidism); only 0.76% showed positive anti-TPO titers, and the mean urinary iodine was 354 *μ*g/L [36].

A community-based cross-sectional study was recently conducted to determine the urinary iodine levels among the school-age population in Popayán-Cauca. Other factors were also evaluated, including the development of eating habits and salt intake, prevalence of goiter, thyroid function and thyroid autoimmunity parameters (anti-TPO titers, anti-thyroglobulin antibodies (anti-Tg), and TSH receptor antibodies (TRAb)), and intelligence quotient (IQ). 100% of the study population used salt in their food, with >99% of the salt used being iodized. The average salt intake per person per day was 18.13 g. Some level of goiter was documented in 37.9% of the school children; the IQ measurement showed that 36.1% of the school children were “mentally weak”. The mean urinary iodine was 510.3 *μ*g/L (80.7% of the subjects had concentrations >300 *μ*g/L, and 87.1% had concentrations >200 *μ*g/L). Positive anti-TPO was found in 42.75% of the subjects but only 2.87% presented positivity for anti-Tg, and 3.62% were positive for both anti-TPO and anti-Tg; none of the subjects were TRAb positive. Finally, an association was identified between the levels of urinary iodine (≥200 *μ*g/L) and the positive anti-TPO titers [37]. 10% of the individuals had TSH levels >4.0 mIU/L (Table 4).

## 5. Monitoring and Evaluation of IDDs Control Programs

Considering that all health interventions must be monitored and their compliance ensured, it is reasonable to expect that USI programs require an effective monitoring and evaluation system [38]. Based on this idea, particular questions, indicators, and topics have been suggested for the assessment of IDD prevention programs. The questions to be asked are as follows: Does the salt produced or imported meet the iodization requirements in the country? Do the countries at risk of iodine deficiency have access to properly iodized salt and is it used in their population? Is there any population group unable to access iodized salt and hence requires care? What is the impact of salt iodization and of other interventions on the iodine status of the population? And finally, what is the relative contribution of iodine intake from various sources?

**Figure 1:**
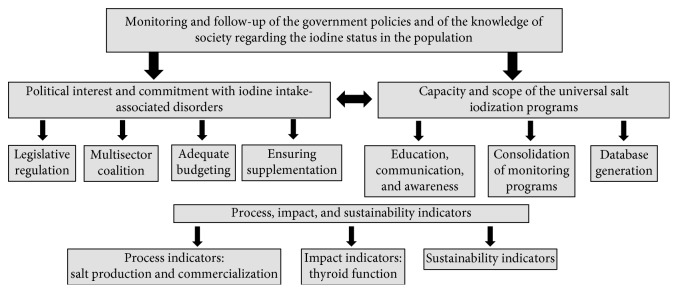
Summary of guidelines and decisions to consider regarding iodine intake programs.

Meanwhile, in countries where the IDD eradication programs have not yet been implemented, the questions are different and include the following: Have IDDs been eliminated as a public health problem? What is the prevalence of IDDs among specific population groups, such as pregnant women and children? And, what are the population-targeted steps required to control IDDs? Along the same lines, three indicators have been described (process, impact, and sustainability) that reflect the monitoring of iodine content in salt, the effect of salt iodization on the population, and the successful elimination of IDDs [39, 40]. Likewise, the following are some of the topics to consider in the programs evaluating DDIs: commitment of the political agencies, adequate management of education and communications programs, consolidation of monitoring systems, and setting of the supplementation objectives. The integration of these indicators, topics, and questions facilitates the development of programs aimed at eradicating IDDs in the most effective and efficient possible manner (Figure 1). Consequently, some program indicators have been described for the sustained elimination of IDDs [41, 42]. It is believed that when at least eight of those indicators are complied with, the IDDs eradication goals will then be met (Table 5).

## 6. Analysis of the Iodine Status in the Population and Its Clinical Impact

The key strategy to achieve a sustained elimination of IDDs is still USI. The international regulatory agencies have recommended the complementary use of iodine supplements (iodized oil) as a temporary measure when iodization cannot be implemented promptly [43–45]. According to the 2004 report of the global database on iodine deficiency (by the World Health Organization (WHO)), five countries had excessive iodine intake (Brazil, Chile, Ecuador, Liberia, and the Republic of Uganda). However, according to the 2017 report of the group of countries with excess iodine intake from the Global Iodine Network (GIN), those countries that had been classified as having excessive iodine intake in 2004 had remarkably lower intake levels 13 years later [46–48]. According to the 2004 WHO report, countries such as Armenia had an adequate intake, while Benin, Colombia, Costa Rica, Honduras, and Qatar reported a more than adequate intake; currently, Uganda continues as the only country with excessive iodine intake after the 2004 WHO report (Tables 6 and 7).

Colombia produces around 540,000 tons of salt annually, a level of production that ensures some degree of “self-sufficiency;” hence, when considering the possibility of changing the levels of iodine in salt, the underlying premise is that the current per capita intake of salt in the country is, on average, 10 g/person/day. However, an important consideration is that the standard established for Colombia regarding iodine concentration in salt (50–100 ppm) is valid at the level of processing plants and packaging companies, since, at home, the established amount is of 15 ppm (when assuming, for instance, that there is a 20% loss of iodine in salt between the production site and home, plus another 20% loss through cooking). The 1994–1998 study determined that the proportion of salt with a >20 ppm iodine concentration (representing the validated range at the international level at that time) among the samples taken from homes was 83.7%, which dropped to 68.3% among the samples analyzed in 1998. The sentinel studies in 1999 and in 2001–2002 found that 73.6% and 90.4% of the samples taken from homes exhibited contents >25 ppm, whilst 81.8% and 98.6% had >15 ppm, respectively. In any case, the proportion of samples with inadmissible home levels dropped, and therefore the indicator was met, since >90% of the homes used effectively iodized salt.

It must be kept in mind that in order to supply 150 *μ*g/day iodine (through salt), the concentration at the production site should be within the range of 20–40 mg of iodine/kg of salt (20–40 ppm, equivalent to 34–66 mg of potassium iodate/kg of salt). When all the salt used in processed foods is iodized, the recommendation is to choose the lowest limit of concentration (20 mg). Under these circumstances, the mean iodine concentration varies between 100 and 200 *μ*g/L. Therefore, if the indicator in the evaluation of IDD reduction establishes that <50% of the iodization means shall be <100 *μ*g/L and that <20% of the iodization means shall be <50 *μ*g/L (so that the mean iodization should be >100 *μ*g/L), Colombia will be fully compliant with the indicator (in accordance with the 1994–1998 study and the three sentinel studies conducted). However, the country is far above these values, as found in the most recent studies (Tables 4 and 7).

Consequently, if the salt intake levels are maintained and the concentration of iodine in salt was set at 20–40 ppm, a “sufficient” supply of iodine would be provided to the population. Furthermore, if the salt intake were to be lowered to <5 g/person/day, this would be consistent with an iodine concentration of around 40 ppm. Nevertheless, since salt is added to commercially manufactured foods prior to selling and since there is a growing substitution of home-cooked meals with discretional use of iodized salt (salt is added at the table and during cooking), it is important that commercial foods contain the proportional amount of iodine required to meet the needs of the population. This is why several international organizations have recommended an iodine concentration in salt of 20–40 ppm at the production site (assuming an average intake of salt per capita of 10 g/person/day) and issued the recommendation of an average salt intake (from all food sources) of <5 g/person/day, in order to reduce the intake of sodium to <2 g/person/day [49, 50]. Furthermore, food salt is also considered a risk factor associated with the development of chronic illnesses other than thyroid disease (for instance, gastric cancer, osteoporosis, kidney stones, and high blood pressure, with a subsequent risk of death and associated comorbidities (ischemic heart disease, stroke, hypertensive nephropathy, inter alia)). It has been estimated that if food salt is lowered to the above-recommended levels, the nationwide prevalence of high blood pressure in Colombia could decrease by 30% (a mild blood pressure decrease reduces the mortality rates from stroke and ischemic heart disease; consequently, if the overall intake of food salt is reduced by at least 15% over 10 years, 8 to 9 million deaths could be prevented) [50–53]. Considering two of the most recent studies conducted in Colombia, which determined the average intake of salt from food to be 11.42 g (the 1994–1998 study) and 18.13 g (2015 study), it is evident that the documented national iodine excess is the result of an excess salt intake with food, and hence all possible strategies shall be implemented to reduce the salt intake in the population [54].

Along these lines, other exogenous forces that are having an impact on the excess of iodine in the Colombian population may not be ruled out; however, considering the two studies that to this date have assessed the average salt intake, it is clear that if you only take into account the average salt intake (household use), it ranges between 14 and 15 g/day (this average intake would be much higher when considering other food sources with a high content of iodine, such as seafood, cereals, eggs, and fortified wheat flour). There are no studies available in Colombia assessing the content of iodine in food and the impact it could have on the health of the population. The mineral content table (year 2015) established that the average content of iodine in greens and vegetables is 0.80 g per 100 g (the iodine content was not identified in other food sources). Therefore, in the case of Colombia, one could say that most of the excess iodine among the population is due to salt for human consumption (based on three main factors: *easy access to iodized salt, amount of iodine added to salt, and excessive salt intake*). An additional risk may be the result of food products, since no other public health policies are in place in Colombia to prevent iodine deficit in the population (for instance, the use of iodized oil or iodization of water, inter alia). Similar results have been found in neighboring countries such as Peru, where excessive iodine has been documented in the population (with adequate iodine concentrations in salt for human consumption). Hence, the excessive level of iodine in the population is the result of the use of iodized salt and iodine-rich foods [55–57].

It was not until the mid-1960s that the prevalence of goiter in Colombia was reduced to <5% (until then, the prevalence had been >30%, with subsequent variations upon the implementation of the USI programs; these studies were limited to measuring the prevalence of goiter, but failed to measure the urinary iodine levels). More recent studies have established the prevalence of goiter, the levels of urinary iodine, the titers of anti-TPO, anti-Tg, and TRAb, among other parameters, and have shown that there is an excessive iodine intake. Only one study showed a “more than adequate” intake and another did not identify urinary iodine levels ≥100 *µ*g/L, which may account for the high rate of goiter and the high prevalence of positivity for anti-TPO and anti-Tg (autoimmune thyroid disease). It may then be considered that the high rates of goiter in Colombia have been due to two extreme levels of iodine intake. Prior to the implementation of USI programs in Colombia, iodine deficiency was the most common iodine-related condition, but after the implementation excess intake rose, along with the rates of goiter.

Additionally, the only study conducted in Colombian pregnant women to date failed to identify an increase in positive anti-TPO status but showed a prevalence of goiter of >25% in this population (this high rate of goiter shall obviously be considered a serious public health problem that calls for immediate action and interventions by the respective health authorities). This low prevalence of anti-TPO positivity may be accounted for, at least partially, by nutritional factors (selenium, zinc, copper, manganese, and vitamin D intake) that could modulate the secretion of these antibodies. It is also worth noting that the studies evaluating intake of iodine based on the geographical area (urban vs. rural) have shown that the intake in urban areas has been consistently higher than that in rural areas, and that rural areas have both “insufficient” and “excess” levels documented. These differences may be explained by a broad variety of factors, including the growing poverty levels of the rural areas, the lack of follow-up and control of USI programs, the use of noniodized salt, or the low iodine concentration of the soils where these populations live. Colombia is predominantly a mountainous country, with particular soil characteristics that in many cases are far from the coastal regions, explaining at least partially the differences in iodine levels, particularly in the rural areas; other related factors may be the presence of goiter-promoting substances in the diet and the exposure to pesticides [58–60].

## 7. Conclusion

The health impact of both a deficit or excess of iodine in the diet requires a balanced approach between the guidelines of the USI programs and the strategies aimed at reducing salt intake. The two strategies to consider involve decreasing the concentration of iodine in salt (20–40 ppm) and reducing the average salt intake (<5 g/day). Both approaches are compatible, profitable, and greatly beneficial for the population. The necessary and relevant partnerships must be established to make these objectives consistent. Furthermore, the authorities responsible for the implementation and surveillance of USI programs must adjust the levels of iodine in salt. These recommendations have shown that the intake of salt can be reduced without jeopardizing the efforts to fortify with micronutrients, particularly in the areas where salt intake is more than adequate or excessive and where such intervention explicitly met the objectives of reducing the rates of goiter, but led to excessive salt intake and higher risk of developing other complications.

## Figures and Tables

**Table 1:** Health consequences of iodine intake deficiency or excess.

Iodine intake	
Deficit	Excess
Hypothyroidism	Hyperthyroidism
Goiter and nodular thyroid disease	Increased risk of thyroid autoimmunity
Increased risk of thyroid cancer	Iodine-induced hyperthyroidism (Jod–Basedow effect)
Increased susceptibility of thyroid to nuclear radiation	Iodine-induced hypothyroidism (Wolff–Chaikoff effect)
Increased rate of abortions and fetal, perinatal, and child mortality	Goiter
Endemic cretinism, growth retardation, decreased intellectual, and labor capacity	Probable rise in cardiovascular mortality

**Table 2:** Epidemiological criteria for the evaluation of iodine nutrition in a population, based on the mean urinary iodine levels, on the range of urinary iodine concentration, or both.

Iodine in women and children		
Intake amount (*µ*g/L)	Intake status	Nutritional contribution
Pregnant women		
<150	Insufficient	N/A
150–249	Adequate	N/A
250–499	More than adequate	N/A
≥500	Excess^*∗*^	N/A

Lactating mothers^*∗∗*^		
<100	Insufficient	N/A
≥100 *µ*g/L	Adequate	N/A

Children <2 years of age		
<100	Insufficient	—
≥100	Adequate	—

School-age children		
<20	Insufficient	Severe iodine deficiency
20–49	Insufficient	Moderate iodine deficiency
50–99	Insufficient	Mild iodine deficiency
100–199	Adequate	Optimal
200–299	More than adequate	Risk of iodine-induced hyperthyroidism in susceptible populations
>300	Excess	Risk of detrimental health consequences (hyperthyroidism, iodine-induced autoimmune thyroid disease)

N/A: no information available from the evaluation table of the United Nations for the nutritional contribution of iodine in pregnant and lactating women. ^*∗*^Exceeding the amount required to prevent and control an iodine deficit. ^*∗∗*^Levels of mean urinary iodine are lower than the iodine requirements due to iodine excretion in breast milk.

**Table 3:** Studies evaluating IDDs in Colombia before its declaration as an IDD-free country.

Parameters evaluated^*∗*^	Year						
	1948	1952	1960	1965	1977–1980	1984–1986	1993
Geographical area	U and R	U and R	U and R	Mainly R	U and R	U and R	U and R
Population	School children	School children	General	School children	General	General	General
Goiter^*∗∗*^	53%	33%	M: 39.5%, F: 43.7%	<2%	<2%	U: 13.5%, R: 52%	15%

F: female; IDD: iodine deficiency disorder; M: male; NR: not reported; U: urban; R: rural. ^*∗*^Urinary iodine was not reported for any year. ^*∗∗*^Prevalence determined by palpation.

**Table 4:** Studies evaluating IDDs in Colombia after its declaration as an IDD-free country.

Parameters evaluated	Year							
	1994–1998	1999	2001–2002	2006–2007	2010	2011	2013–2014	2015
Geographical area	U	U and R	U and R	U and R	U and R	U	U	Mainly U
Population	School children (*n*=19, 530)	School children (*n*=NR)	School children (*n*=1380)	School children (*n*=444)	School children (*n*=128)	Adults (*n*=437)	Pregnant women (*n*=392)	School children (*n*=140)
Urinary iodine, mean in *µ*g/L	≥100 (in >90% of the population)	380 (U: 705, R: 148)	415 (U: 430, R: 380)	272.4 (U: 285.5, R: 72.6)	401.2	424.2	354	510.3
Goiter^*∗*^	6.5%	NR	NR	NR	88%	NR	25.5%	37.9%
Anti-thyroid antibodies^*∗∗*^	NR	NR	NR	NR	Anti-TPO: 4%	Anti-TPO: 28.9%	Anti-TPO: 0.76%	Anti-TPO: 42.75%; anti-Tg: 2.87%; anti-TPO and anti-Tg: 3.62%; TRAb: 0%

Anti-Tg: anti-thyroglobulin antibodies; anti-TPO: anti-thyroid peroxidase antibodies; IDD: iodine deficiency disorder; NR: not reported; R: rural; TRAb: TSH receptor antibodies; U: urban. ^*∗*^Prevalence determined by palpation. ^*∗∗*^Prevalence determined by immunoassay.

**Table 5:** Programmatic indicators for the sustained elimination of IDDs.

Indicator	Function
Establishing a multisector coalition	Implement a national program for the elimination of IDDs. All the stakeholders (including the salt industry) shall be represented with responsibilities; stakeholders must meet at least twice a year.
Evidence of a political commitment	IDDs inclusion in the national budget.
Legislation and regulations	Enact laws and regulations supporting the universal salt iodization strategy
Evaluation of IDDs progress	Establish methods for progress evaluation in eliminating IDDs through programs evaluating progress every 3 years.
Maintenance of adequate laboratories	Maintain access to adequate laboratories that provide accurate data on iodine levels in salt, in the urine and in thyroid (via function test).
Development of education and social promotion programs	Establish an education and social mobilization program through information strategies on the importance of iodine for the population.
Availability of iodine in salt data	Maintain a constant and routine availability of the iodine content data by having the values available from factories (at least on a monthly basis) and at the home level (at least every 5 years).
Availability of urine iodine data	Maintain the availability of the population-based data on the value of urinary iodine (at least every 5 years).
Commitment of the salt industry	Demonstrate the ongoing cooperation of the salt industry, determined by the maintenance of quality control measures and reducing the costs of iodide and iodate.
Database generation	Maintain a database at the national level to keep a log of the regular monitoring results, including home-based population coverage, urinary iodine values, and results of thyroid function tests when available.

IDDs: iodine deficiency disorders.

**Table 6:** Countries with excessive iodine intake in the general population^*∗*^.

Country	Total population, in year 2002	Urinary iodine, median in *µ*g/L	Year of survey	Population, age in years
Brazil	176,257,000	360	2000	School children (6–12)
Chile	15,613,000	984	2001	School children (6–18)
Ecuador	12,810,000	420	1999	School children
Liberia	3,239,000	321	1999	School children (6–11)
Republic of Uganda	25,004,000	310	1999	School children (6–12)

^*∗*^According to the global database on iodine deficiency (WHO, 2004).

**Table 7:** Countries with excess iodine intake in the general population and in pregnant women^*∗*^.

Country	Total population, in 2015	Urinary iodine, median in *µ*g/L	Year of survey	Population, age in years	Urinary iodine in pregnant women, median in *µ*g/L, year of survey (status)
Democratic Republic of Armenia	3,017,712	313	2005	School children (8–10)	NR
Republic of Benin	10,879,829	318	2011	School children (6–12)	NR
Republic of Colombia	48,228,704	415	2002	School children	354, 2013–2014 (more than adequate intake)
Republic of Costa Rica	4,807,850	314	2008–2009	School children	NR
Republic of Djibouti	887,861	335	2015	School children	265, 2015 (more than adequate intake)
Democratic Republic of Georgia	3,999,812	321	2005	School children (6–12)	NR
Republic of Honduras	8,075,060	356	2005	School children	NR
State of Qatar	2,235,355	341	2014	School children (6–12)	NR
Salomon Islands	583,591	328	2007	School children (6–12)	NR
Federal Republic of Somalia	10,787,104	417	2009	School children	NR
Republic of Uganda	39,032,383	464	2005	School children (6–12)	NR
